# Optimization of Ultrasound-Assisted Extraction of Phenolic Compounds from *Annona muricata* By-Products and Pulp

**DOI:** 10.3390/molecules24050904

**Published:** 2019-03-05

**Authors:** Gabriela Aguilar-Hernández, María de Lourdes García-Magaña, María de los Ángeles Vivar-Vera, Sonia Guadalupe Sáyago-Ayerdi, Jorge Alberto Sánchez-Burgos, Juliana Morales-Castro, Luis Miguel Anaya-Esparza, Efigenia Montalvo González

**Affiliations:** 1 Laboratorio de Integral de Investigación de Alimentos, Tecnológico Nacional de México/Instituto Tecnológico de Tepic, Av. Tecnológico 2595, Lagos del Country, Tepic 63175, Mexico; gaby.mca2017@gmail.com (G.A.-H.); mgarciam@ittepic.edu.mx (M.d.L.G.-M.); ssayago@ittepic.edu.mx (S.G.S.-A.); jorgealberto_sanchezburgos@yahoo.com.mx (J.A.S.-B.); l_m_ae@hotmail.com (L.M.A.-E.); 2 Tecnológico Nacional de México/Instituto Tecnológico de Tuxtepec. Depto. de Ingeniería Química y Bioquímica-Maestría en Ciencias en Alimentos, Av. Dr. Víctor Bravo Ahuja S/N. Col. 5 de Mayo, Tuxtepec 68350, Mexico; maryangeles.vivar@gmail.com; 3 Departamento de Ingenierías Química y Bioquímica, Tecnológico Nacional de México/Campus-Instituto Tecnológico de Durango, Felipe Pescador 1830 Ote., Durango 34080, Mexico; jmorales@itdurango.edu.mx

**Keywords:** soursop fruit, phenolic compounds, pulp and by-products, ultrasound-assisted extraction

## Abstract

Ultrasound-assisted extraction (UAE) is widely used; however, the efficiency of extraction depends on the raw materials. Therefore, optimization of UAE must be investigated for each type of plant material. By-products from soursop fruit have not been studied as a source of bioactive compounds. In this work, the optimization of UAE conditions (extraction time (5, 10, and 15 min), pulse cycle (0.4, 0.7, and 1 s), and sonication amplitude (40%, 70%, and 100%)) for the extraction of phenolic compounds (soluble, hydrolyzable, condensed tannins, and total polyphenols) from soursop by-products (seed, peel, and columella) and pulp was evaluated using response surface methodology. The optimal conditions for UAE to obtain the highest total polyphenol content from by-products and pulp was dependent on the raw material. Peel resulted in the highest content of total polyphenols (187.32 mg/g dry matter [DM]) followed by columella (164.14 mg/g DM), seed (36.15 mg/g DM), and pulp (33.24 mg/g DM). The yield of polyphenolic content from peel and columella obtained with UAE was higher (32–37%) than conventional extraction for 2 h under stirring (14–16%). The contents of gallic acid (0.36–15.86 µg/g DM), coumaric acid (0.07–1.37 µg/g DM), and chlorogenic acid (9.18–32.67 µg/g DM) in the different parts of the fruit were higher in the extracts obtained by UAE compared with a conventional extraction method (0.08–0.61, 0.05–0.08, 3.15–13.08 µg/g DM, respectively), although it was dependent on the raw materials. Soursop by-products can be functionally important if they are used to extract bioactive compounds by UAE; a technology with high potential for commercial extraction on a large scale.

## 1. Introduction

Soursop (*Annona muricata* L.) belongs to the family Annonaceae, and it is found in different tropical and subtropical areas worldwide. Its components (stems, leaves, and roots) and some parts of the fruit (pulp and seeds) have been used medicinally against human diseases such as cancer, diabetes, cardiovascular and anti-inflammatory problems, and parasitic infections [1,2].

The consumption of soursop fruits has increased around the world due to its importance for health [3]. The soursop fruit presents an irregular form (typically heart-shaped) that is dark green when unripe and pale green when ripe. The fruits measure 20 to 25 cm in length and 10 to 12 cm in diameter, and weigh measure from 0.25 to 5 kg. The surface of the fruit is covered with small conical protuberances [3,4]. Soursop fruit consist of 67% edible components, and it is principally consumed as fresh fruit, although in an increasing percentage is processed as juice, nectar, and puree; however, 33% of the whole fruit is discarded as waste (20% peel, 8.5% seeds, and 4% columella) [3]. As a result, there is a high level of by-products, especially peels and seeds; amounts of 660 kg of by-products/day at processing plants could result in local pollution [5]. Soursop leaves, pulp, and seeds have been recognized as sources of valuable bioactive compounds such as polyphenols, alkaloids, and acetogenins [2,6,7]. Soursop by-products such as columella and peel have not been studied. In some studies, it has been reported that many fruit by-products may contain higher levels of bioactive compounds than the edible parts [8,9]. Therefore, soursop by-products could be considered as a potential source to extract natural bioactive compounds with antioxidant activity or pharmaceutical applications.

Phenolic compounds are strong antioxidants, although their protective action goes beyond the modulation of oxidative stress; thus, they have been used as nutritional supplements, additives in functional foods, pharmaceutical products to prevent chronic diseases such as cardiovascular diseases, type 2 diabetes, and some types of cancers [9]. The polyphenolic extract has antioxidant activity by different mechanisms and was shown to inhibit the pro-inflammatory enzymes (cyclooxygenase, lipoxygenase, and phospholipase A_2_) in metabolic syndrome, oxidative stress, and inflammatory processes [10]. Furthermore, it has been reported that polyphenols act primarily through modulation of the PI3K/AKT pathway, demonstrating that the polyphenols are inflammatory mediators in an in vitro colon cancer model [11].

Traditionally, phenolic compounds from plant materials have been extracted by conventional extraction methods (extraction with stirring, maceration, cold pressing, squeezing, and hydro-distillation). However, most of these techniques are based on the extracting power of different solvents along with the application of heat and long extraction times. These approaches can cause loss of polyphenol content due to oxidation, ionization, and hydrolysis [12,13,14]. Rasheed et al. [14] evaluated different extraction methods (decoction, infusion, and maceration) and their effect on the bioactive compound profile of *Hibiscus sabdariffa* (roselle) extracts. Cold maceration proved to be better for preserving anthocyanins, whereas the infusion method was more suitable for recovering organic acids. In that context, the impact of the extraction method plays an important role on the extractable compounds and their related biological activities [14].

As an alternative technology, ultrasound (US) is being used increasingly. Ultrasound may produce a cavitation effect, the action of which can cause physical and mechanical changes in raw materials facilitating extraction of compounds [15,16]. US has been applied for polyphenolic extraction from muicle leaves [16], starfruit leaves [13], as well as pomegranate [17] and orange [18] peel, among many other plants with nutritional or pharmaceutical effects [9]. Anaya-Esparza et al. [16] obtained a higher phenolic content (54 mg/g) using ultrasound-assisted extraction (UAE, 2 min extraction time and 0.7 s pulse cycle at 55% of sonication amplitude) than with stirring (46.4 mg/g, 2 h using a magnetic stirrer at room temperature) and thermal decoction (47.7 mg/g, boiled at 60 °C for 5 min) in muicle leaves. UAE can improve the extraction rate, reduce the extraction time, and increase the stability of compounds compared with conventional extraction methods such as stirring and thermal decoction [16]. Although there is a lot research on UAE in vegetal material, the optimization of US extraction on soursop pulp or by-products has not been investigated; the extraction efficiency depends on the UAE conditions and raw materials. Therefore, the optimization of UAE must be investigated for each type of plant material [19].

The Box-Behnken design (BBD) tool is used for optimization of extraction procedures of biological compounds and is an important response surface methodology (RSM) [20]. BBD is a rotatable second-order design based on a three-level incomplete factorial design. This design is used extensively as an economic method for extracting a large amount of information with a small number of experiments [21]. On the other hand, RSM allows the interaction of the independent variables with the response variables to be monitored using a collection of statistical and mathematical methods [20,21]. The aim of this study was to evaluate the effect of UAE conditions on the extraction of phenolic compounds from soursop seed, peel, columella, and pulp and to use RSM to optimize the UAE parameters (extraction time, pulse cycle, and sonication amplitude) for extracting polyphenolic compounds from pulp and by-products of soursop fruits using BBD. In addition, the optimal UAE conditions are compared with a conventional extraction method for extraction of polyphenolic compounds and their yield.

## 2. Results and Discussion

### 2.1. Ultrasound-Assisted Extraction of Soluble Polyphenols from Soursop Samples

The experimental data for soluble polyphenols (SP) obtained by UAE from soursop peel, columella, seeds, and pulp are shown in Table 1. Statistical differences (*p* < 0.01) were observed between treatments (experimental conditions: X_ET_ = extraction time (5, 10, and 15 min), X_PC_ = pulse cycle [0.4, 0.7, and 1 s], and X_SA_ = sonication amplitude [40%, 70%, and 100%]) and raw materials (seeds, peel, columella, and pulp). The extraction of SP from soursop pulp and by-products was dependent on the experimental conditions. The highest SP contents from peel were 157.40 and 156.29 mg/g DM in samples treated for 5 and 15 min (X_PC_, 0.7 s; X_SA_, 100%), respectively. Under the same experimental conditions (X_ET_ = 5 min, X_PC_ = 0.7 s, X_SA_ = 100%), the highest SP content in seeds was obtained (28.38 mg/g DM), and comparable values were observed in powder seeds after 10 min of exposure at 100% sonication amplitude (X_SA_) and 0.7 s pulse cycle (X_PC_). In contrast to peel and seeds, the highest SP values for columella (143.35 mg/g DM) and pulp (23.62 mg/g DM) were observed when the lowest values for the experimental conditions were applied (X_ET_ = 10 min, X_PC_ = 0.4 s, X_SA_ = 40% and X_ET_ = 5 min, X_PC_ = 0.7 s, X_SA_ = 40%, respectively). León-Fernández et al. [22] reported an SP content of 35.6 mg/g (DM) in soursop pulp extract after applying extraction with an ultrasonic bath (3 h, 42 kHz at 25 °C) and using methanol as solvent. In this work, a 11-fold reduction in extraction time was achieved compared with the report of León-Fernández et al. [22]. Several studies have shown that UAE could be used as an efficient method for the extraction of polyphenolic compounds from plant material [13,16].

Other authors have argued that the mechanical effects generated by UAE modify the morphology of the cell walls, and a more porous surface is obtained, thus facilitating the extraction [23]. Thus, the components in the free state migrate from the material to the solution. In addition, the ultrasonic treatment accelerates the molecular motion of the solution and hence helps to quickly and effectively combine the components with the solution [24].

On the other hand, peel and columella showed higher (*p* < 0.01) SP content than pulp and seeds after UAE. These results may be attributable to the composition and complexity of each matrix and because the peel protects the fruit from external factors, and the columella is a conduit of nutrients and protects the seeds [25]. Similar trends were previously reported by Albuquerque et al. [26] who reported higher phenolic content in peel (1.7–1.9 g/kg) than in pulp (0.30–1.20 g/kg) and seeds (0.30–0.40 g/kg) from *Annona cherimoya* Mill.

**Figure 1:**
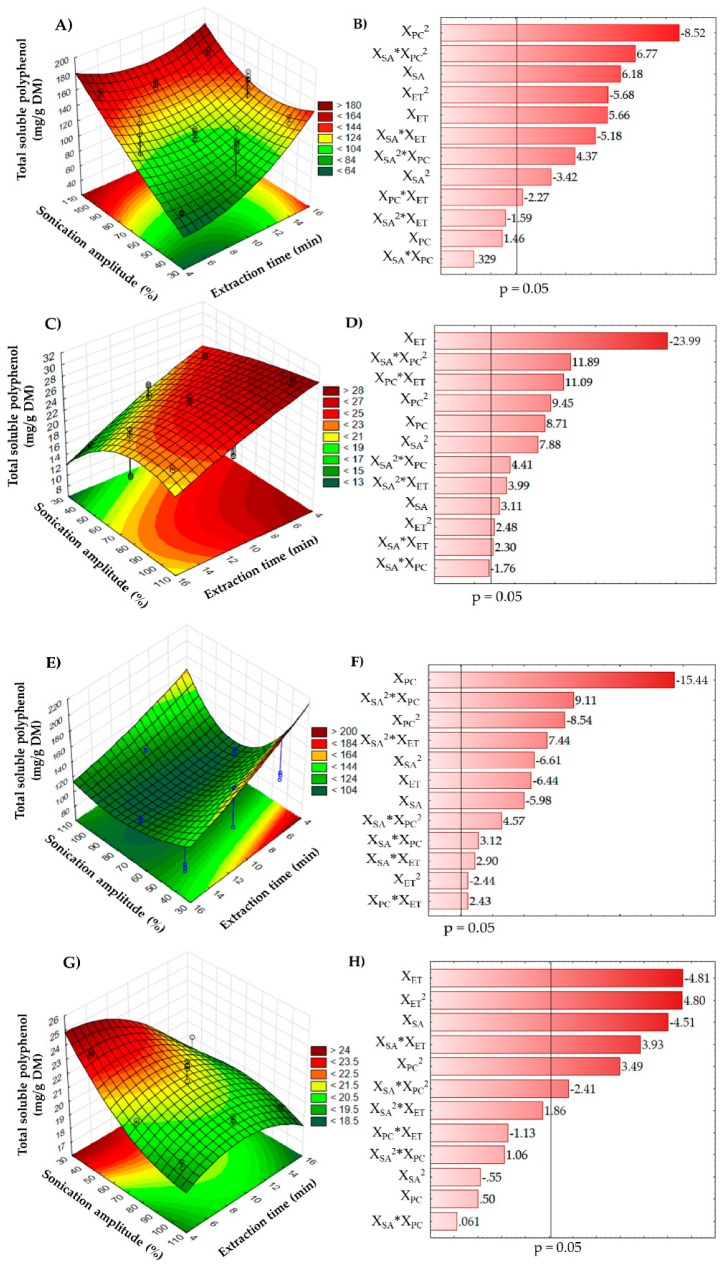
Response surface plots and Pareto charts for soursop peel (**A**,**B**), seeds (**C**,**D**), columella (**E**,**F**), and pulp (**G**,**H**) showing the effect of ultrasound-assisted extraction on the soluble polyphenol content. DM, dry matter; X_ET_, exposition time (min); X_SA_, sonication amplitude (%); X_PC_, pulse cycle.

The significant interaction effects (*p* < 0.01) of the UAE parameters on the SP content in peel (Figure 1A), seeds (Figure 1C), columella (Figure 1E), and pulp (Figure 1G) are shown in three-dimensional response surface plots, where the shapes contour plots (elliptical form) indicate the interactions between the corresponding variables [27]. Results indicated that SP extraction by UAE was observed in the whole experimental domain and in all vegetal material studied, independent of the extraction time, pulse cycle, and sonication amplitude used. However, the highest SP content from peel (Figure 1A) and seeds (Figure 1C) can be obtained at 15 and 5 min, respectively, with medium pulse cycles (0.7 s) and sonication amplitude (100%). Conversely, the highest SP values in columella (Figure 1E) and pulp (Figure 1G) can obtained at lower experimental conditions (X_ET_ = 7.5 min, X_PC_ = 0.4 s and X_ET_ = 5 min, X_PC_ = 0.7 s, respectively), in particular for the sonication amplitude (X_SA_, 40%). In addition, the Pareto charts (Figure 1B,D,F,H) show the effect of independent variables on the extraction of SP at a confidence level of 95%. The effect (negative or positive) of the variables on SP extraction is dependent on each matrix, and the effect of the independent variables (and their interactions) on each vegetal material could be ranked as follows: for pulp, X_ET_ > X_SA_ > X_PC_; peel, X_PC_ > X_SA_ > X_ET_; columella, X_PC_ > X_SA_ > X_ET_; and seeds, X_ET_ > X_PC_ > X_SA_. Xu; and Pan [28] reported that extraction efficiency for all-trans-lycopene from red grapefruit is a function of the independent variables (extraction time, temperature, solvent/material ratio, ultrasonic intensity, and duty cycle). As in this study, the UAE conditions for SP compounds from soursop samples were dependent on the plant matrix [25].

RSM for the extraction of SP from soursop pulp, peel, columella, and pulp by UAE as a function of extraction time, pulse cycle, and sonication amplitude was developed using a multiple regression technique. A second-order polynomial equation (Table 2) for each material was obtained, which described the effect of the test variables as well as the combined effect of all test variables on the response [21]. The analysis of variance of the SP content showed that the experimental data from pulp, columella, peel, and seeds have good correlation (R^2^ = 0.77, R^2^ = 0.94, R^2^ = 0.91, and R^2^ = 0.97, respectively), and thus, adequate adjustment of the experimental data to the model was observed (lack of fit, *p* > 0.05), except for pulp, which showed weak adjustment (R^2^ = 0.77) (Table 2). The lack of fit demonstrated the fitness of the model, giving an indication of approximation to the real system. Comparable results were reported by Bashi et al. [29] during optimization (extraction temperature, pH, liquid/solid ratio, and extraction time) of UAE of phenolic compounds from *Achillea biebersteinii* using RSM (R^2^ = 0.85).

Some coefficients (Table 3) were not significant (*p* > 0.05) to the model: for peel, X_ET_ and X_SA_^2^*X_ET_, in pulp, X_SA_, X_ET_, X_SA_*X_PC_, X_SA_^2^*X_PC_, and X_SA_^2^*X_ET_. On the other hand, all coefficients for seed and columella were significant (*p* < 0.05) to the model.

Pan et al. [16] mentioned that when UAE is applied in plant materials (e.g., pomegranate peel) the intensity level and pulse duration has a prominent effect on the polyphenolic extraction yield; and a long extraction time had a negative effect. However, the authors mentioned that efficacy of treatment depended on the matrix composition and the cellulose-hemicellulose-lignin ratio. In addition, a slight increase in temperature from 1 to 2.88 °C (Table 1) for all UAE runs was observed in a normal reaction when UAE was applied as a consequence of interaction of cavitation and extraction time. This temperature increase can be also attributable to heat transference from cavitation bubbles, which may cause a gradual temperature increase in the medium and facilitate extraction of the compounds. Similar trends were reported previously during UAE (extraction time, 2 min; pulse cycle, 0.7 s; sonication amplitude, 55%) of polyphenolic compounds from muicle leaf extracts [16]. However, there was no drastic increase in temperature because the temperature was controlled during the experiments.

### 2.2. Evaluation of Model Reliability and Comparison of Ultrasound-Assisted Extraction with Conventional Extraction

The optimum UAE conditions for SP extraction from *A. muricata* peel, seeds, columella, and pulp are shown in Table 4. To verify the reliability of the model, treatments were performed using the optimal conditions for each sample as recommended by Aydar et al. [19]. The experimental values of the SP content (Table 5) for all samples were in agreement with the predicted values under the optimal conditions (see Table 4). Therefore, an efficient extraction of polyphenols from soursop samples using UAE was possible, as has been reported by other studies where RSM was used to optimize the extraction of polyphenols by UAE from pomegranate [17], orange [18] peel, muicle [16], and starfruit leaves [13].

The SP content from peel and columella, hydrolyzable polyphenol (HP) content from peel and seeds, and condensed tannin (CT) content from pulp were significantly higher (*p* < 0.01) when UAE was used compared with conventional extraction (Table 5). However, there was no significant difference (*p* > 0.01) in the SP content from seeds and pulp, HP content from columella and pulp, or CT content from peel, seeds, or columella for both extraction methods; however, the extraction time was significantly reduced to 45–55 min. Also, the total polyphenol content was higher using UAE in all samples except pulp, and the yield of phenolic content from peel and columella was higher under UAE (32.34–37.51%) than for organic aqueous extraction (14.87–16.68%). Cvjetko Bubalo et al. [30] reported an increase (more than 30%) in the extraction of SP in grape peel when the samples were treated by UAE (15 min, 60 W, 20 °C) compared with conventional extraction. Velásquez-García et al. [31] added a pretreatment with ultrasound in the extraction of bioactive compounds from mesocarp of chontaduro (*Bactris gasipaes*) using supercritical CO_2_, which did not increase the extraction yield, but the operation time was shorter than for conventional extraction. Sousa et al. [32] found that UAE was inefficient for extracting ellagitannins. These authors mentioned that this can be justified because the UAE process produces a large amount of energy, which may lead to cleavage or oxidation of molecules, mainly those with a higher molecular mass.

The effect of ultrasound is attributed to interaction with the plant material, altering its physical and chemical properties, and to a cavitation effect, which facilitates the release of extractable compounds and enhances mass transport by disrupting the plant cell walls [15]. Our results are in agreement with Jovanović et al. [33], who compared UAE and maceration extraction methods for extraction of polyphenols from *Thymus serpyllum* L. herb. They reported that the polyphenolic yield obtained by UAE was higher (22.81 mg/L) than the maceration extraction method (18.12 mg/L), and indicated that UAE could be used for extraction of polyphenols in the future due to the high yield of polyphenolic and flavonoid content, short extraction time, and low degradation of the extracted compounds compared with other extraction methods.

### 2.3. Soluble Polyphenols Profile

A total of nine phenolic compounds (gallic, coumaric, cinnamic, caffeic, chlorogenic, protocateic, 4-hydroxybenzoic, syringic, and neochlorogenic acids) were identified (Table 6). The type of polyphenol and their concentration were dependent on the raw material and the extraction method, but it was clear that the UAE led to the highest concentration of some polyphenolic compounds such as gallic, coumaric, and chlorogenic acids compared with conventional extraction. Extraction under conventional techniques is usually characterized by poor quality of extracts because thermodegradation of bioactive compounds is induced [34]; UAE is an attractive and green alternative. However, phenolic profile from seeds was not different (*p* < 0.01) using both methods, probably because the SP content from seeds was very low.

## 3. Materials and Methods

The experimental work was carried out in two stages. In the first stage, the optimal experimental conditions were determined to extract the highest SP content from soursop pulp and by-products under UAE by using RSM. The second stage consisted of evaluation of the accuracy of the model by measuring the SP, HP, and CT contents, total polyphenols, and the profile of the polyphenolic compounds obtained under optimal UAE conditions. The yield obtained with UAE versus conventional extraction was compared.

### 3.1. Plant Material and Chemicals

Soursop fruits (*Annona muricata* L.) were collected in orchards located in Compostela, Nayarit, Mexico, at the consumption maturity stage (15–19 °Brix). The pulp and by-products (peel, seeds, and columella) were separated manually. The samples were freeze-dried (Labconco 77522020, Kansas City, MO, USA) at −50 °C under pressure of 0.12 mbar. The dried samples were then ground in a food processor (Nutribullet NB-101B, Los Angeles, CA, USA) and sieved using a stainless steel mesh (no. 35; Fisher Scientific, Hampton, NH, USA) to a particle size of 500 µm. Phenolic standards, Folin-Ciocalteu phenol reagent, methanol, water, and acetic acid of HPLC grade were purchased from Sigma-Aldrich (St Louis, MO, USA).

### 3.2. Experimental Design

A BBD was used to determine the optimal UAE conditions for extracting polyphenols from soursop fruit pulp and by-products with three levels for each factor. The goal was to evaluate the individual and interactive effects of extraction time (X_ET_, min), pulse cycle (X_PC_, s), and sonication amplitude (X_SA_, %) at 40%, 70%, and 100% on SP content. A randomized experimental order was used to reduce the effect of unexplained variability on the observed response. Table 1 shows the experimental design used in the study; the three independent variables were studied at three levels: X_ET_ (5, 10, and 15 min), X_PC_ (0.4, 0.7, and 1.0), X_SA_ (40%, 70%, and 100%).

### 3.3. Ultrasound-Assisted Extraction Procedure

The extraction of polyphenolic compounds from soursop pulp and by-products was performed using a UP400S ultrasonic system (400 W, 24 kHz frequency) (Hielscher Ultrasonics, Teltow, Germany). Extraction solutions were according to Pérez-Jiménez et al. [35]. The ultrasonic probe (H7 Tip 7, Hielscher, Teltow, Germany) with 100% maximum amplitude corresponded to 175 μm and acoustic power density of 300 W/cm^2^. The ultrasonic probe was submerged 2 cm into the extraction solution. The procedure started by weighing the lyophilized samples (0.5 g), placing them in an extraction tube with 20 mL of a water-methanol solution (50:50 *v*/*v*) acidified to pH 2 with HCl (0.8% *v*/*v* 16 mM HCl was added), and then the samples were subjected to UAE (according to the experimental design; see Table 1). A recirculating cold water bath (Thermo Scientific 2870, Waltham, MA, USA) was used to maintain the extraction temperature in a range of 25 ± 2 °C. The samples were centrifuged (Hermle Z32HK, Wehingen, Germany) at 8000× *g* for 10 min at 4 °C, the supernatants were recovered, and the residues were resuspended in 20 mL of acetone–water solution (70:30 *v*/*v*). The samples were processed again under the same controlled UAE conditions and centrifuged. The supernatants were combined and analyzed to measure SP.

### 3.4. Soluble Polyphenolic Compounds

The SP content was determined using Folin–Ciocalteu reagent following the Montreau procedure [36] with slight modifications. The supernatants (250 µL) were mixed with 1250 µL of Folin-Ciocalteu reagent and incubated in tubes for 5 min. Sodium carbonate solution (1000 µL, 75 g/L) was then added and the tubes were incubated in a water bath for 15 min at 50 °C. Then 270 µL were transferred into the wells of a 96-well microplate reader (Synergy HT Multi-Detection Microplate Reader, BioTek Instruments, Winooski, VT, USA), and the absorbance was measured at 765 nm. SP content was calculated with a standard curve (R^2^ = 0.998) for gallic acid, and the results were expressed as milligrams of gallic acid equivalents per gram of sample of dry matter (mg/g DM). Each standard and sample was analyzed in triplicate.

### 3.5. Response Surface Methodology Analysis

Once the data was obtained (SP), RSM was applied to obtain the optimal processing conditions for the four samples. A second-order polynomial equation derived from RSM was used to calculate the predicted response (Equation (1)).

(1) $$Y=\beta 0+\sum_{i=A}^{E}\beta \mathrm{iXi}+\sum_{i=A}^{E}\sum_{j=A\neq i}^{E}\beta \mathrm{ijXi}+E$$

where Y is the predicted response (SP), Xi are the uncoded or coded values for the factors (X_ET_, X_PC_, and X_SA_), β0 is a constant, βi are the main effect coefficients for each variable, and βij are the interaction effect coefficients. Model adequacy was evaluated by analysis of variance (ANOVA) to determine the effects of significant interactions in the model (*p* < 0.01) and by quantification of the coefficient of determination (R-squared and R-adjust).

### 3.6. Model Reliability and Comparison of Ultrasound-Assisted Extraction with Conventional Extraction

The optimal UAE conditions by RSM analysis for extracting polyphenolic compounds from soursop pulp and by-products were used to verify the accuracy of the model. The content of SP, HP, and CT were measured, and the total polyphenols and the yield were calculated. In addition, the profile of the polyphenolic compounds was determined by HPLC. The results for phenolic compound using the optimal UAE conditions were compared with the results with conventional extraction.

#### 3.6.1. Conventional Extraction of Polyphenols

Organic aqueous extraction was performed with 0.5 g of lyophilized samples in 20 mL of methanol-water solution (50:50, *v*/*v*) acidified with HCl (0.8% *v*/*v* 16 mM HCl). The mixture was stirred at room temperature (25 ± 2 °C) at a moderate speed (10× *g*) for 1 h in a shaker (Heidolph Rex 2, Heidoplh Instruments, Schuwabach, Germany). Then the extracts were centrifuged at 8000× *g* for 10 min at 4 °C. The supernatants were recovered and the residues were resuspended in 20 mL of an acetone-water solution (70:30, *v*/*v*); the samples were stirred for 1 h at room temperature and centrifuged under the same conditions. The method of extraction was according Pérez-Jiménez et al. [35]. The supernatants were combined and used to measure SP; the residues were used to measure HP and CT.

#### 3.6.2. Soluble Polyphenols, Hydrolyzable Polyphenols, and Condensed Tannins

Polyphenols were extracted under optimal UAE conditions or conventional extraction. Supernatants (soluble polyphenolic extracts) and residues were obtained. SPs were measured in the supernatants following the methodology described earlier. The residues were obtained in two lots: one lot was used to evaluate HP and the other to measure CT.

HPs were determined with the method described by Hartzfeld et al. [37]. Hydrolysis of the samples (0.5 g) with 20 mL methanol/concentrated H_2_SO_4_ (90:10 *v*/*v*) at 85 °C for 20 h was performed. The samples were then centrifuged (10 min, 25 °C, 5000× *g*). The supernatants were saved and the residues were resuspended in distilled water twice, and the mixture was centrifuged at each dilution. The supernatants (extracts) were combined. The extracts (500 µL) were mixed with 500 µL of Folin-Ciocalteu reagent and incubated for 5 min at room temperature. Then, 1000 µL of sodium carbonate solution (75 g/L) was added to the mixture and incubated in a water bath for 15 min at 50 °C. Subsequently, absorbance was measured at 765 nm. HPs were calculated using a standard curve for gallic acid. The results were reported as mg/g DM. CTs were determined in the residues according to Reed et al. [38]. Ten milliliters of butanol/HCl/ FeCl_3_ (97.5:2.5 *v*/*v*) solution was added to the residues (0.5 g) and incubated at 100 °C for 3 h. The samples were centrifuged (10 min, 4 °C, 6000× *g*) and CTs were determined in the supernatants. Absorbance was measured at 555 nm using a spectrophotometer (Jenway 6705, Dunmow, UK). CTs were calculated from a standard curve of proanthocyanidins from Mediterranean carob pods (*Ceratonia siliqua* L.). The CT content was expressed as mg/g DM.

#### 3.6.3. Polyphenol Profile by HPLC

The partial identification of phenolic acids was performed in the SP extracts under optimal UAE conditions and with conventional extraction. SP extracts were concentrated in a rotary evaporator and resuspended in 1 mL of eluent A. The samples (20 μL) were injected into an HPLC system (Agilent Technologies 1260 Infinity, Waldbronn, Germany) equipped with a photodiode array detector and a C18 reverse-phase column (5 μm particle size, 4.6 mm in diameter, 250 mm long; Thermo Scientific, Sunnyvale, CA, USA). The mobile phases were acidified water with 2% acetic acid (eluent A) and acidified water (0.5% acetic acid)–methanol (10:90, eluent B). Standards and samples were analyzed using a gradient program: 0% B; 0–35 min, 35% B; 35–55 min, 75% B; 55–60 min, 100% B; 60–70 min; 0% B, at a flow rate of 0.4 mL/min. The peak areas of the samples were detected at 280 and 320 nm, and a calibration curve (0.5–300 μg/mL) of gallic, chlorogenic, caffeic, cinnamic, caffeic, chlorogenic, protocateic, syringic, neochlorogenic, 4-hydroxybenzoic, and *p*-coumaric acids was used to identify and quantify the polyphenols [7]. Total phenolic compounds were determined as the sum of SP, HP, and CT.

#### 3.6.4. Yield of Polyphenolic Compounds

Yield was defined as the percentage of the total extracted polyphenols (SP, HP, and CT) from the total weight of the sample (g). The extraction yield was calculated (Equation (2)) as suggested by Aydar et al. [19].

(2) $$\mathrm{Yield}\;\left(\%\right)=\frac{\mathrm{Total}\;\mathrm{polyphenols}\;\left(\mathrm{mg}\right)}{\mathrm{Sample}\;\left(g\right)}\times 100$$

### 3.7. Statistical Analysis

Data were expressed as means ± standard deviation (*n* = 6). In the first stage, data were analyzed with RSM. In the second stage, the experiments were conducted in a one-factorial experimental design. The analyses were performed using ANOVA (*p* < 0.01) with STATISTICA v. 10 (Statsoft, Tulsa, OK, USA) software. Fisher’s least significant difference method was used to examine the differences between means (α = 0.01).

## 4. Conclusions

The optimal UAE conditions are dependent on the type of raw material. The results in this study confirmed that short extraction times using UAE were sufficient to achieve swelling of the cells, leading to increased permeability of vegetal cell walls, and more phenolic compounds could be extracted from soursop in decreasing order of peel followed by columella, seeds, and pulp. The efficiency of UAE was higher than the conventional extraction method. The polyphenolic profile of the extracts showed the presence of nine polyphenolic compounds including gallic, coumaric, cinnamic, caffeic, chlorogenic, protocateic, 4-hydroxybenzoic, syringic, and neochlorogenic acids. The content of phenolic acids was higher for UAE than for conventional extraction. The present study highlights that by-products (peel and columella) from soursop fruits could be a potential source of polyphenolic compounds and that UAE is an efficient technology to extract these bioactive compounds.

## Figures and Tables

**Table 1:** Effect of process variables of ultrasound-assisted extraction on soluble polyphenols from peel, seeds, columella, and pulp from soursop fruit.

Run	UAE Conditions			Final Temperature (°C)	Soluble Polyphenols (mg/g Dry Matter)			
	X_ET_	X_SA_	X_PC_		Peel	Seed	Columella	Pulp
1	5	40	0.7	23.38 ± 1.84	74.26 ± 0.84 ^i,B^	24.27 ± 0.18 ^c,d,C^	115.01 ± 4.76 ^b,A^	23.62 ± 0.16 ^a,C^
2	15	40	0.7	22.25 ± 2.72	126.45 ± 8.42 ^e,f,A^	16.19 ± 0.42 ^h,C^	84.06 ± 1.30 ^f,g,B^	20.00 ± 0.69 ^e,f,C^
3	5	100	0.7	24.88 ± 1.89	157.40 ± 6.03 ^a,A^	28.38 ± 0.98 ^a,C^	106.59 ± 3.93 ^c,B^	19.50 ± 0.66 ^f,g,C^
4	15	100	0.7	24.50 ± 1.08	156.29 ± 4.14 ^a,b,A^	21.99 ± 0.54 ^f,C^	91.67 ± 2.53 ^d,e,B^	19.26 ± 0.47 ^g,C^
5	5	70	0.4	22.25 ± 2.10	116.07 ± 7.90 ^f,g,A^	25.07 ± 0.58 ^b,c,B^	106.44 ± 5.55 ^c,A^	19.54 ± 0.19 ^f,g,B^
6	15	70	0.4	23.38 ± 2.32	141.68 ± 5.25 ^b,c,d,A^	15.85 ± 0.22 ^h,C^	105.82 ± 5.05 ^c,B^	19.24 ± 0.11 ^g,C^
7	5	70	1	24.75 ± 1.55	154.51 ± 9.22 ^a,b,c,A^	24.91 ± 0.64 ^b,c,C^	91.64 ± 0.21 ^d,e,B^	20.62 ± 0.29 ^d,C^
8	15	70	1	24.63 ± 2.43	156.76 ± 5.71 ^a,A^	23.81 ± 0.58 ^d,e,C^	104.48 ± 2.22 ^c,B^	19.35 ± 0.34 ^g,C^
9	10	40	0.4	22.88 ± 1.93	135.92 ± 2.35 ^d,e,A^	20.85 ± 0.61 ^g,B^	143.35 ± 1.52 ^a,A^	21.45 ± 0.62 ^c,B^
10	10	100	0.4	24.50 ± 1.08	141.50 ± 6.60 ^c,d,A^	20.29 ± 0.52 ^g,C^	116.47 ± 0.36 ^b,B^	20.42 ± 0.77 ^d,e,C^
11	10	40	1	24.88 ± 1.31	129.23 ± 3.30 ^d,e,f,A^	23.11 ± 0.52 ^e,C^	91.08 ± 0.17 ^d,e,B^	21.33 ± 0.27 ^c,C^
12	10	100	1	25.13 ± 1.65	138.20 ± 8.03 ^d,e,A^	21.26 ± 0.76 ^f,g,C^	81.46 ± 1.86 ^f,g,B^	20.40 ± 0.18 ^d,e,C^
13	10	70	0.7	23.88 ± 0.75	110.39 ± 7.12 ^g,h,A^	25.39 ± 0.50 ^b,C^	93.66 ± 1.13 ^d,B^	22.19 ± 0.58 ^b,C^
14	10	70	0.7	24.63 ± 1.03	99.18 ± 0.87 ^h,A^	24.45 ± 0.55 ^b,c,d,C^	86.74 ± 0.59 ^e,f,B^	22.63 ± 0.38 ^b,C^
15	10	70	0.7	24.63 ± 1.25	103.26 ± 7.24 ^g,h,A^	24.21 ± 0.87 ^c,d,C^	78.53 ± 0.51 ^g,B^	19.83 ± 0.49 ^e,f,g,C^

UAE, ultrasound-assisted extraction; X_ET_, exposition time (min); X_SA_, sonication amplitude (%); X_PC_, pulse cycle. Data are expressed as means ± standard deviation (*n* = 3). Different lowercase letters indicate significant statistical differences between treatments (α = 0.05); different capital letters indicate significant statistical differences between raw materials (α = 0.05).

**Table 2:** The predicted mathematical model for the extraction of soluble polyphenols (mg/g) from *Annona muricata* peel, seeds, columella, and pulp after ultrasound-assisted extraction.

Sample	Polynomial Equation	R^2^
Peel	594.18 − 8.08X_SA_ + 0.04X_SA_^2^ − 1459.15X_PC_ + 891.37X_PC_^2^ + 4.51X_ET_ + 0.61X_ET_^2^ + 21.09X_SA_*X_PC_ − 9.12X_SA_*X_PC_^2^ − 0.06X_SA_^2^*X_PC_ − 0.27X_SA_*X_ET_ + 0.00X_SA_^2^*X_ET_ − 3.89X_PC_*X_ET_	0.91
Seeds	74.23 − 0.98X_SA_ + 0.004X_PC_^2^ − 109.02X_PC_ + 59.83X_PC_^2^ − 2.40X_ET_ − 0.01X_ET_^2^ + 2.15X_SA_*X_PC_ − 1.14X_SA_*X_PC_^2^ − 0.004X_SA_^2^*X_PC_ + 0.035X_SA_*X_ET_ − 0.001X_SA_^2^*X_ET_ + 1.35X_PC_*X_ET_	0.97
Columella	778 − 14.68X_SA_ + 0.08X_SA_^2^ − 906.85X_PC_ + 367.59X_PC_^2^ − 21.45X_ET_ + 0.14X_ET_^2^ + 14.32X_SA_*X_PC_ − 3.30X_SA_*X_PC_^2^ − 0.06X_SA_^2^*X_PC_ + 0.47X_SA_*X_ET_ − 0.003X_SA_^2^*X_ET_ + 2.24X_PC_*X_ET_	0.94
Pulp	24.40 − 0.27X_SA_ + 0.002X_SA_^2^ + 35.55X_PC_ − 27.78X_PC_^2^ − 0.11X_ET_ − 0.04X_ET_^2^ − 0.21X_SA_*X_PC_ + 0.27X_SA_*X_PC_^2^ − 0.001X_SA_^2^*X_PC_ + 0.02X_SA_*X_ET_ − 0.001X_SA_^2^*X_ET_ − 0.16X_PC_*X_ET_	0.77

X_SA_, sonication amplitude (%); X_PC_, pulse cycle (s); X_ET_, extraction time (min); R^2^, regression coefficient.

**Table 3:** Regression coefficients of predicted quadratic polynomial models with the ultrasound-assisted extraction conditions on the soluble phenolic content from *Annona muricata* peel, seeds, columella, and pulp.

Source	Regression Coefficients			
	Total Soluble Phenolic Content β Coefficient			
	Peel	Seed	Columella	Pulp
Mean/intercept	594.18	74.23	778.00	20.48
X_SA_	−8.08 ^+^	−0.98 ^+^	−14.68 ^+^	−0.29 ^++^
X_SA_^2^	0.04 ^+^	−0.004 ^+^	0.08 ^+^	0.002 ^+^
X_PC_	−1459.15 ^+^	−109.02 ^+^	−906.85 ^+^	35.55 ^+^
X_PC_^2^	891.37 ^+^	59.83 ^+^	367.59 ^+^	−27.78 ^+^
X_ET_	4.51 ^++^	−2.40 ^+^	−21.45 ^+^	−0.11 ^++^
X_ET_^2^	0.61 ^+^	−0.01 ^+^	0.14 ^+^	−0.04 ^+^
X_SA_*X_PC_	21.09 ^+^	2.15 ^+^	14.35 ^+^	−0.21 ^++^
X_SA_*X_PC_^2^	−9.12 ^+^	−1.14 ^+^	−3.30 ^+^	0.27 ^+^
X_SA_^2^*X_PC_	−0.06 ^+^	−0.004 ^+^	−0.06 ^+^	−0.001 ^++^
X_SA_*X_ET_	−0.27 ^+^	0.03 ^+^	0.47 ^+^	0.023 ^+^
X_SA_^2^*X_ET_	0.00 ^++^	−0.0002 ^+^	−0.003 ^+^	−0.0001 ^++^
X_PC_*X_ET_	−3.89 ^+^	1.35 ^+^	2.24 ^+^	−0.05 ^++^
R-square	0.91	0.97	0.94	0.77
R-adjust	0.87	0.96	0.92	0.69

X_ET_, extraction time; X_PC_, pulse cycle; X_SA_, sonication amplitude. ^+^ Significant (*p* < 0.05); ^++^ non-significant (*p* > 0.05).

**Table 4:** Optimal conditions of ultrasonic-assisted extraction on soluble polyphenols from *Annona muricata* peel, seed, columella, and pulp.

Parameter	Peel	Seeds	Columella	Pulp
Extraction time (min)	15	5	7.5	5
Pulse cycle (s)	0.4	0.7	0.4	0.7
Sonication amplitude (%)	99.95	100	40	40
Optimal response (mg/g DM)	161.76	28.38	153.65	23.62
−95% Confidence limit	148.21	27.63	147.64	22.74
+95% Confidence limit	175.30	29.13	159.65	24.50
Confidence interval (±)	27.09	1.5	11.98	1.76

−95% confidence limit, lower limit; +95% confidence limit, upper limit; confidence interval, difference between upper and lower limits; DM, dry matter.

**Table 5:** Soluble and hydrolyzable polyphenols, condensed tannins, total polyphenols, and yield from *Annona muricata* peel, seed, columella, and pulp using the optimal conditions for ultrasound-assisted extraction and conventional extraction (extraction for 2 h under stirring, see Section 3.6.1).

Parameter	Ultrasound-Assisted Extraction				Conventional Extraction			
	Peel	Seeds	Columella	Pulp	Peel	Seeds	Columella	Pulp
Soluble polyphenols (mg/g dry matter)	171.26 ± 8.83 ^a^	24.92 ± 0.33 ^d^	152.97 ± 1.01 ^b^	24.70 ± 1.52 ^d^	133.96 ± 0.03 ^c^	23.58 ± 2.14 ^d^	124.91 ± 8.81 ^c^	24.76 ± 1.72 ^d^
Hydrolyzable polyphenols (mg/g dry matter)	8.40 ± 0.14 ^c^	11.05 ± 0.38 ^a^	6.14 ± 0.96 ^d^	5.68 ± 0.21 ^e^	6.51 ± 0.23 ^d^	9.01 ± 0.41 ^b^	7.31 ± 0.14 ^d^	5.47 ± 0.28 ^e^
Condensed tannins (mg/g dry matter)	7.66 ± 0.74 ^a^	0.18 ± 0.02 ^d^	5.13 ± 0.20 ^b^	2.86 ± 0.22 ^c^	8.21 ± 0.49 ^a^	0.72 ± 0.07 ^d^	4.90 ± 0.69 ^b^	0.91 ± 0.02 ^d^
Total polyphenols (mg/g dry matter)	187.32 ± 3.23 ^a^	36.15 ± 0.24 ^d^	164.14 ± 0.70 ^b^	33.24 ± 0.65 ^e^	148.68 ± 0.25 ^c^	33.31 ± 0.87 ^e^	137.12 ± 3.21 ^c^	31.14 ± 3.01 ^e^
Polyphenolic yield (%)	37.51 ± 2.87 ^a^	7.13 ± 0.14 ^c^	32.34 ± 0.31 ^a^	6.59 ± 0.25 ^c^	16.68 ± 0.15 ^b^	6.67 ± 0.48 ^c^	14.87 ± 1.43 ^b^	5.42 ± 1.01 ^c^

All values are means ± standard deviation of three determinations. Different letters in each column indicate significant statistical differences between treatments (α = 0.01). Experimental conditions for ultrasound-assisted extraction: (1) peel: X_ET_ = 15 min, X_PC_ = 0.4 s, X_SA_ = 40%; (2) seeds: X_ET_ = 5 min, X_PC_ = 0.7 s, X_SA_ = 100%; (3) columella: X_ET_ = 7.5 min, X_PC_ = 0.4 s, X_SA_ = 40%; (4) pulp: X_ET_ = 5 min, X_PC_ = 0.7 s, X_SA_ = 40%.

**Table 6:** Profile of soluble polyphenols from *Annona muricata* peel, seed, columella, and pulp using the optimal conditions of ultrasound-assisted extraction and conventional extraction (extraction for 2 h under stirring, see Section 3.6.1).

No.	Compound	Retention Time (min)	Ultrasound-Assisted Extraction (μg/g Dry Matter)				Conventional Extraction (μg/g Dry Matter)			
			Peel	Seed	Columella	Pulp	Peel	Seed	Columella	Pulp
1	Gallic acid	20.66	14.50 ± 0.44 ^a^	0.36 ± 0.01 ^c^	12.16 ± 0.37 ^b^	15.86 ± 1.68 ^a^	1.10 ± 0.08 ^c^	0.46 ± 0.03 ^c^	0.61 ± 0.12 ^c^	0.08 ± 0.01 ^c^
2	Coumaric acid	46.61	1.37 ± 0.09 ^a^	0.07 ± 0.00 ^c,d^	0.08 ± 0.01 ^bc^	0.07 ± 0.01 ^c,d^	0.15 ± 0.04 ^b^	0.07 ± 0.02 ^c,d^	0.08 ± 0.01 ^b,c^	0.05 ± 0.01 ^c,d^
3	Cinnamic acid	52.91	45.51 ± 1.88 ^a^	40.48 ± 1.21 ^a,b^	30.36 ± 1.98 ^b^	42.04 ± 8.72 ^a,b^	37.16 ± 1.33 ^a,b^	42.51 ± 0.59 ^a^	14.51 ± 3.88 ^c^	7.67 ± 1.14 ^c^
4	Caffeic acid	37.17	43.68 ± 1.78 ^a^	32.62 ± 1.22 ^b^	nd	nd	30.20 ± 8.38 ^b^	34.44 ± 0.46 ^b^	11.83 ± 3.19 ^c^	nd
5	Chlorogenic acid	34.23	32.67 ± 0.53 ^a^	12.33 ± 0.46 ^b,c^	9.18 ± 0.59 ^c^	12.80 ± 2.72 ^b^	12.25 ± 2.87 ^b,c^	13.08 ± 0.18 ^b^	nd	3.15 ± 0.25 ^d^
6	Protocateic acid	23.02	150.46 ± 6.62 ^a^	133.47 ± 5.02 ^a^	nd	nd	123.12 ± 4.28 ^a^	140.97 ± 1.62 ^a^	nd	25.37 ± 3.77 ^b^
7	4-Hydroxybenzoic acid	31.16	145.98 ± 6.47 ^a^	nd	94.45 ± 6.15 ^a,b^	131.63 ± 2.87 ^a,b^	117.37 ± 32.65 ^b^	nd	45.96 ± 12.43 ^c^	24.22 ± 3.60 ^c,d^
8	Syringic acid	41.62	883.71 ± 3.94 ^a^	780.77 ± 9.31 ^b^	nd	nd	nd	824.05 ± 1.89 ^b^	nd	148.83 ± 2.14 ^c^
9	Neochlorogenic acid	24.05	78.86 ± 3.48 ^a^	69.70 ± 2.62 ^a^	51.90 ± 3.38 ^b^	72.32 ± 1.31 ^a^	nd	73.56 ± 0.97 ^a^	nd	13.30 ± 1.98 ^c^

All values are means ± standard deviation of three determinations. Different letters in each column indicate significant statistical differences between treatments and between samples (α = 0.01). nd, not detected.
